# The Effect of Intracoronary Stem Cell Injection on Markers of Leukocyte Activation in Acute Myocardial Infarction

**DOI:** 10.14740/cr375w

**Published:** 2015-02-09

**Authors:** Ragnhild Helseth, Trine Opstad, Svein Solheim, Ketil Lunde, Harald Arnesen, Ingebjorg Seljeflot

**Affiliations:** a Center for Clinical Heart Research, Department of Cardiology, Oslo University Hospital Ulleval, Oslo, Norway; b Faculty of Medicine, University of Oslo, Oslo, Norway; c Department of Cardiology, Oslo University Hospital Rikshospitalet, Oslo, Norway

**Keywords:** Bone marrow stem cells, Stem cell treatment, Acute myocardial infarction, Neutrophil markers, Pentraxin 3, Myeloperoxidase

## Abstract

**Background:**

Beneficial effects of stem cell treatment during acute myocardial infarction (AMI) have been suggested, but the effects on inflammation are controversial. The neutrophil cell markers pentraxin 3 (PTX3) and myeloperoxidase (MPO) are both reported to be elevated during AMI. We studied the effects of stem cell treatment in ST-elevation myocardial infarction (STEMI) on PTX3 and MPO levels.

**Methods:**

Subjects with STEMI undergoing percutaneous coronary intervention (PCI) were randomized to intracoronary injection of bone marrow cells (mBMCs) (n = 50) or controls (n = 50). Blood samples were drawn 1 day before mBMC injection (baseline), after 1 day, 3 days, 2 - 3 weeks and 3 months. Enzyme-linked immunosorbent assay (ELISA) and RT-PCR were used for biochemical analysis. Myocardial necrosis was quantified by single photon emission computed tomography (SPECT) and creatine kinase MB (CKMB) levels.

**Results:**

PTX3 and MPO levels did not differ between the groups at any time points. Within the mBMC group, overall changes in both variables were observed (P < 0.01), with decreased levels from baseline throughout. Within the control group, similar patterns were observed. The relative reduction of PTX3 from baseline to day 1 was significantly less pronounced in the mBMC group compared to controls (P = 0.002), whereas no differences in relative changes from baseline were observed for MPO. Plasma and gene expression levels of PTX3 in leukocytes correlated significantly at all time points (r = 0.379 - 0.448, P < 0.01, all). MPO was significantly correlated to baseline left ventricular ejection fraction (LVEF) (r = -0.229, P = 0.025) and peak CKMB (r = 0.200, P = 0.05).

**Conclusions:**

Stem cell treatment had limited effect on plasma levels of PTX3 and MPO. The initially high PTX3 and MPO levels, the genetic regulation of PTX3 and the association between MPO and myocardial injury support the importance of neutrophil cell activation in STEMI.

## Introduction

Despite standardized acute medical care and early percutaneous coronary intervention (PCI), loss of viable myocardium is still a major clinical issue in acute myocardial infarction (AMI). Although a certain myocardial regeneration is exacerbated in AMI [1-3], this mitotic activity is obviously not adequate to prevent a net loss of viable cardiomyocytes. Administration of bone marrow-derived stem cells (mBMCs) in human subjects with AMI has been suggested to moderately improve left ventricular ejection fraction (LVEF) and reduce infarct size, although conflicting results exist [4]. The mechanism behind the proposed beneficial effect of mBMCs is probably multifactorial, involving paracrine effects through secretion of cytokines and growth factors on cytoprotection, neovascularization and cardiac repair [5, 6].

The number of circulating neutrophil granulocytes has been reported to increase during AMI, and this increase is associated with infarct size, left ventricular function and mortality [7, 8]. Neutrophil granulocytes secrete a varietey of mediators which may link neutrophil cell activation to atherosclerosis, plaque vulnerability and cardiac remodeling [9-11].

Pentraxin 3 (PTX3) is a member of the pentraxin family and is produced by a variety of cells in response to inflammatory stimuli [12]. Levels of PTX3 are reported to be elevated in the early phase of AMI [13-15], and an immediate source of secretion is suggested to be the granules of circulating neutrophils [14, 16]. By displaying both pro- and antiinflammatory effects [12], the exact role of PTX3 in atherothrombosis and subsequent mycardial remodeling is controversial. Its circulating levels during AMI have, nevertheless, been shown to be associated with the risk of new coronary events and mortality [17, 18]. A recent study in mice with subcutaneous transplantation of mesenchymal stem cells in AMI reported improved left ventricular function and increased genetic expression of PTX3, suggesting that part of the cardioprotective effect of stem cell therapy was mediated by PTX3 [19].

The hemeprotein myeloperoxidase (MPO) is secreted from the granules of activated neutrophils, monocytes and macrophages. MPO participates in the atherosclerotic process through stimulated LDL oxidation, endothelial dysfunction and plaque instability [20], and has also been associated with adverse ventricular remodeling [11, 21]. High levels of MPO have been reported in the acute phase of AMI [22] and also to be an independent risk factor for development of cardiovascular disease (CVD) [20]. Administration of stem cells derived from bone marrow, adipose tissue and umbilical cord has in different rat models been reported to reduce MPO activity [23-26].

The present study is a substudy of the autologeous stem-cell transplantation in acute myocardial infarction (ASTAMI) trial [27] investigating the effect of intracoronary administration of mBMCs on PTX3 and MPO levels and the relation between these neutrophil cell markers and myocardial injury. We hypothesized that mBMCs would reduce levels of neutrophil cell markers and that levels of the biomarkers would be associated with myocardial injury.

## Material and Methods

### Subjects and study design

Data presented in this study are based on blood samples obtained from participants in the ASTAMI trial. Details of the ASTAMI study design have been given previously [28]. Briefly, subjects within the age 40 - 75 years (n = 100) with anterior wall ST-elevation myocardial infarction (STEMI) were randomized to intracoronary injection of autologeous mBMC or to a control group without injection. Both groups underwent primary PCI with stent implantation of the left anterior descending artery (LAD). Main exclusion criteria were previous Q-wave infarction, cardiogenic shock or serious comorbidity interfering with protocol compliance.

The ASTAMI trial was approved by the Regional Committee for Medical Research Ethics and all included subjects gave written informed consent for study participation. The study is registered at ClinicalTrials.gov, NCT 00199823.

### Laboratory methods

#### Blood sampling

Blood samples were obtained by standard venipuncture after an overnight fast, before morning medication between 8 am and 9 am. Blood samples were taken at baseline (4 - 5 days after the AMI and 1 day before stem cell treatment) and then after day 1, day 3, 2 - 3 weeks and 3 months.

#### Enzyme immunoassays

PTX3 and MPO levels were determined by commercially available enzyme-linked immunosorbent assays (ELISA) in EDTA plasma (R&D Systems, Abingdon, Oxon, UK and Mercodia AB, Uppsala, Sweden, respectively), prepared within 1 h by 10 min centrifugation at 4 °C at 2,500 × g. Plasma samples were stored at -80 °C prior to analyses. Samples from all time points were measured in the same run in order to avoid bias due to assay variability. The inter-assay coefficients of variation for PTX3 and MPO were 8.5% and 8.9%, respectively.

#### Gene expression analysis

PAXgene blood RNA tubes (Preanalytix Qiagen GmBH, Germany) were collected in a randomly selected subset of patients at all time points (n = 48). RNA isolation was performed by a PAXgene^®^ Blood RNA Kit (PreAnalytix, Qiagen) with an additional cleaning step (RNeasy^®^ MinElute^®^ Cleanup Kit, Qiagen). Total RNA was reversely transcribed into complementary DNA (cDNA) by use of qScript cDNA SuperMix (Quanta Biosciences, Inc., Gaithersburg, USA). PTX3 and MPO mRNA levels were then determined by RT-PCR by the Viia^TM^ 7 Real-Time PCR System (Applied Biosystems, Foster City, CA, USA), normalized to β2-microglobulin (HS99999907_m1, Applied Biosystems) and calculated as relative quantification using the previously described ΔΔCt method [29].

#### Left ventricular function and infarct size assessment

LVEF and size of infarcted LAD perfusion area (%) were obtained by electrocardiogram-gated single photon emission computed tomography (SPECT) (GE Medical Systems) with 4D-MSPECT software at baseline (4.0 ± 1.4 days after the acute event). Myocardial necrosis was additionally quantified by peak levels of creatine kinase MB (CKMB).

### Statistical analysis

As levels of both PTX3 and MPO were skewly distributed, non-parametric statistics were used throughout. Descriptive data are given as mean (standard deviation (SD)), proportions (numbers) and medians (25, 75 percentiles) as appropriate. Intergroup differences were assessed by two-sample *t*-test, Mann-Whitney test for two independent samples or Chi-square test as appropriate. Mann-Whitney test for two independent samples was used to analyze differences in relative change from baseline between the randomized groups. Overall intragroup change was assessed by Friedsman test, followed by Wilcoxon signed-rank test. Correlation analyses were performed using the Spearman rho. P-value of < 0.05 was considered significant. Data analyses were performed using SPSS Statistics, Version 21 and 22.

## Results

### Study population

**Figure 1:**
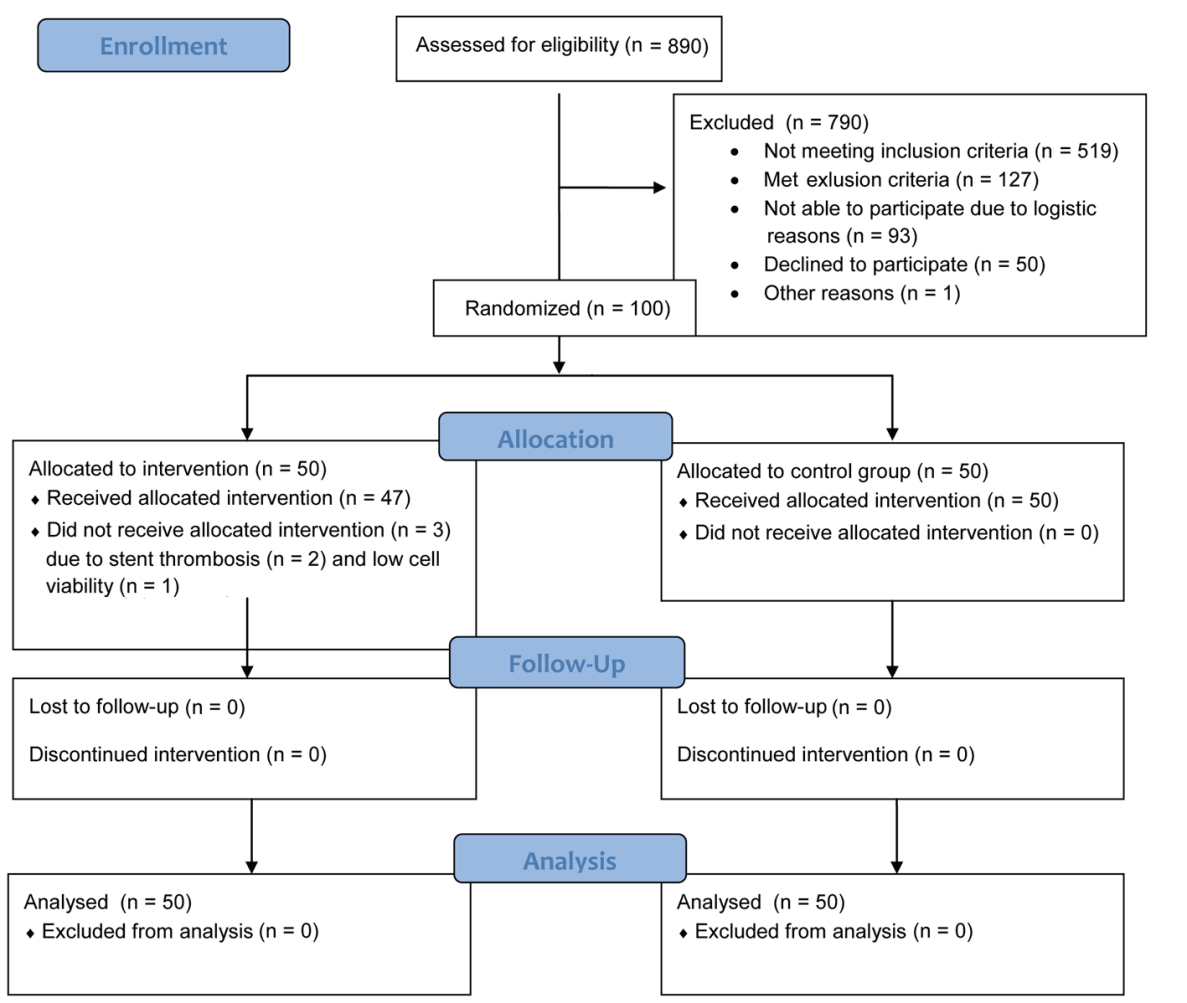
Flow diagram.

In all, 100 patients were randomized to either intracoronary injection of mBMCs (n = 50) or to the control group (n = 50) (Fig. 1). Subject characteristics did not differ significantly between the groups at inclusion (Table 1), and LVEF did not differ significantly between the groups at 6 months [28].

**Table 1:** Characteristics of the Study Population According to the Randomized Groups

	mBMC group (n = 50)	Control group (n = 50)
Age	58.1 (8.5)	56.7 (9.6)
Sex (n of males)	42	42
Hypertension (n)	17	17
Diabetes (n)	5	4
Smokers (n)	20	40
BMI (kg/m^2^)	26.3 (3.9)	27.1 (3.5)
Total cholesterol (mmol/L)	4.4 (3.7 - 5.1)	4.5 (3.8 - 4.9)
LDL cholesterol (mmol/L)	2.9 (2.3 - 3.5)	2.9 (2.4 - 3.3)
HDL cholesterol (mmol/L)	1.0 (0.8 - 1.2)	1.0 (0.9 - 1.3)
Triglycerides (mmol/L)	1.3 (1.0 - 1.7)	1.3 (1.1 - 1.5)
LVEF (%)	43 (35, 48)	44 (34 - 49)
Peak CKMB (ng/L)	400 (220.5 - 447.0)	357 (205.0 - 423.0)
Baseline WBC count (10^9^/L)	8.1 (6.8 - 9.6)	8.4 (7.1 - 8.9)
Baseline neutrophile count (10^9^/L)	4.6 (3.8 - 5.9)	4.8 (4.1 - 5.9)
Medication at discharge		
Aspirin (%)	100	100
Clopidogrel (%)	100	100
ACE-I/ATII antagonist (%)	100	98
Beta blocker (%)	100	100
Statin (%)	100	100
Diuretics (%)	42	32

Values are given as mean (SD), proportions (n), medians (25, 75 percentiles) or percent (%). ACE-I: angiotensin-converting-enzyme inhibitor; ATII antagonist: antiotensin II antagonist; WBC: white blood cell.

### The effect of intracoronary injection of mBMC

Plasma levels of PTX3 and MPO did not differ between the mBMC group and the control group at any time point (Table 2). Relative reduction of PTX3 from baseline to day 1 was less pronounced in the mBMC group compared to the control group (P = 0.002) (Table 2), whereas no between group differences in relative changes from baseline were observed for MPO.

**Table 2:** Plasma Levels of PTX3 and MPO According to the Randomized Groups at Baseline and Follow-Up

	Baseline	Day 1	Day 3	2 - 3 weeks	3 months
PTX3 (ng/mL)					
mBMC group	0.92 (0.67 - 1.38)	0.91^§^ (0.68 - 1.41)	0.84* (0.50 - 1.28)	0.74* (0.45 - 1.31)	0.62* (0.46 - 1.19)
Control group	1.18 (0.71 - 1.33)	0.76* (0.49 - 1.17)	0.73* (0.47 - 1.06)	0.68* (0.42 - 1.10)	0.68* (0.35 - 1.06)
MPO (ng/mL)					
mBMC group	80 (60 - 120)	82* (60 - 106)	79 (57 - 106)	71* (54 - 98)	69* (49 - 92)
Control group	79 (59 - 115)	76 (54 - 101)	75* (61 - 92)	73* (53 - 83)	64* (48 - 88)

Values are given as median (25, 75 pencentiles). ^§^Difference in relative change from baseline (P < 0.05) between the groups. *Change from baseline (P < 0.05) within the respective treatment groups.

Within the mBMC group, an overall change in PTX3 and MPO was observed (P < 0.01 for both), with decreasing levels from baseline to day 3 and further to all time points for PTX3 (P < 0.05 for all) and decreased levels from baseline to day 1, 2 - 3 weeks and 3 months for MPO (P < 0.05 for all). Within the control group, an overall change in PTX3 and MPO was also observed (P < 0.01 for both) with similar patterns as observed within the mBMC group (Table 2).

### Gene expression in leukocytes

**Figure 2:**
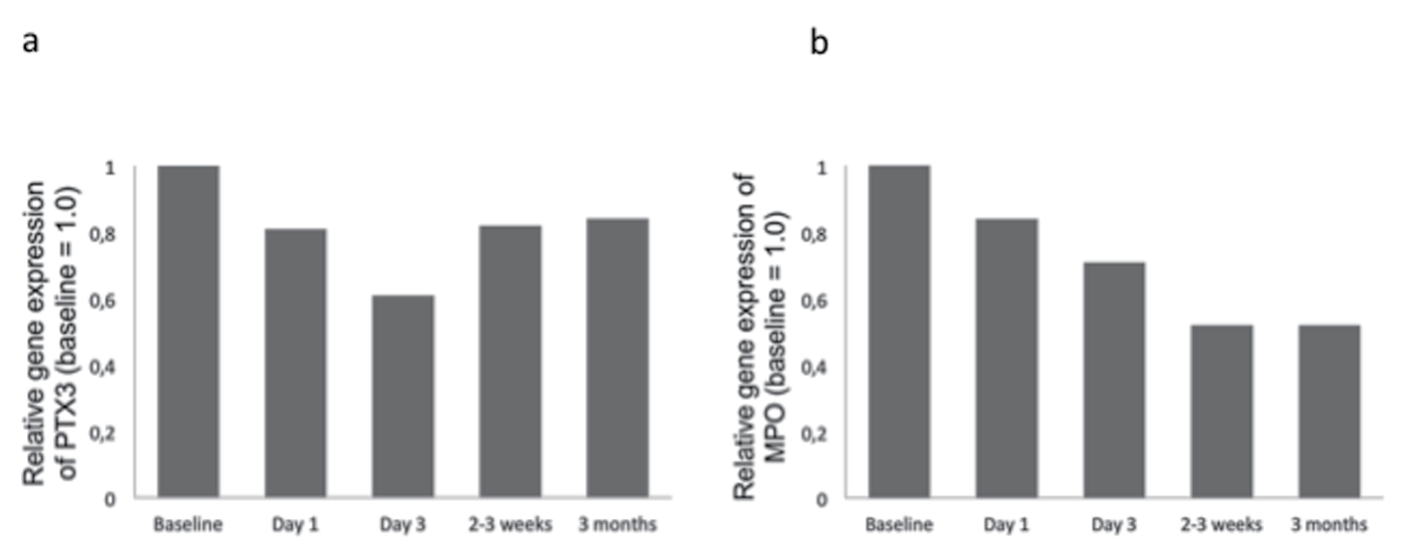
Gene expression of PTX3 (panel a) and MPO (panel b) at follow-up relative to baseline in the randomly selected subset (n = 48) of the total population.

**Figure 3:**
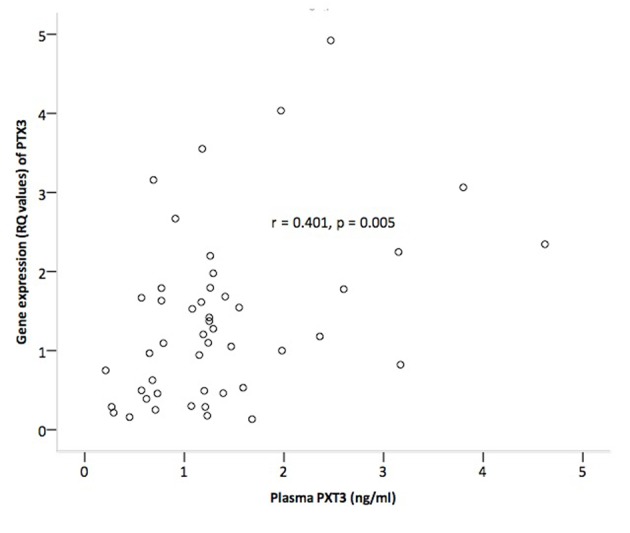
Correlation between plasma levels and gene expression levels of PTX3 in circulating leukocytes at baseline.

Intracoronary injection of mBMCs did not influence gene expression of PTX3 and MPO in circulating leukocytes (n = 48), except from a greater relative reduction in MPO gene expression at 3 months in the mBMC group (P < 0.05, data not shown). In the total subpopulation, the gene expression of PTX3 and MPO decreased from baseline throughout the study period within the range of 16-39% and 16-48%, respectively (Fig. 2). Gene expression of PTX3 correlated significantly to circulating PTX3 levels at all corresponding time points from baseline to 3 months (r = 0.379 - 0.448, P < 0.01 for all). Baseline correlation is shown in Figure 3. No such correlation was observed for MPO.

### Correlations between biomarkers and LVEF, myocardial necrosis and leukocyte count

Baseline PTX3 levels did not correlate to baseline LVEF or infarct size measured by neither SPECT nor peak CKMB. Baseline MPO levels correlated inversely to baseline LVEF (P = 0.025) and positively to peak CKMB (P = 0.05) (Table 3). Baseline neutrophile count correlated to both plasma PTX3 and plasma MPO at day 1 (r = 0.271, P = 0.012 and r = 0.275, P = 0.01, respectively).

**Table 3:** Correlations Between Biomarkers, LVEF and Myocardial Necrosis

	Baseline PTX3		Baseline MPO	
	r	P	r	P
Baseline LVEF^§^	-0.110	0.290	-0.229	0.025
Infarct size*	-0.014	0.892	0.101	0.323
Peak CKMB	0.037	0.720	0.200	0.05

^§^SPECT. *Proportion of LAD perfusion area.

## Discussion

In this substudy of the ASTAMI trial, administration of autologous mBMCs after STEMI had limited effect on PTX3 and MPO levels. However, both leukocyte markers were highest early after the STEMI, indicating transient leukocyte activation. Plasma levels of PTX3 seemed to reflect PTX3 gene expression in leukocytes, and plasma levels of MPO were significantly related to LVEF and myocardial necrosis.

As previously reported from the ASTAMI study and in line with other studies, no improvement of LVEF 6 months after mBMC treatment could be demonstrated [28-32]. In the present study, administration of mBMCs did not affect plasma levels of PTX3 and MPO beyond a smaller relative reduction of PTX3 from baseline to day 1. While mBMC treatment might not influence LVEF, it might stimulate early prolongation of neutrophil activation, the significance of which remains to be explored. To the best of our knowledge, this is the first report on leukocyte activation by mBMCs in humans.

Elevated PTX3 levels have been reported in the acute phase of AMI, peaking around less than 8 h [13, 15] and normalized levels have been reported 48 h after the acute event [14]. MPO levels have been reported to be elevated until 4 h after the acute event with a subsequent decline to 12 h and then a rise after 24 h [22]. A substantial amount of neutrophils has furthermore been reported to be completely depleted of MPO within the first 24 h after symptom onset [33]. Our analyses of PTX3 and MPO from a median of 4 - 5 days after the acute event are probably in a declining phase. Interestingly, we still observed a significant decrease in both markers throughout the study period, indicating prolonged leukocyte activation in the subacute phase of STEMI.

Though mainly not influenced by mBMC, gene expression of PTX3 and MPO in circulating leukocytes decreased throughout the study period suggesting elevated gene expression in the subacute phase of AMI. We observed, however, a strong correlation between gene expression of PTX3 and plasma levels of PTX3 at all corresponding time points. These observations indicate that the PTX3 measured in the circulation to a major degree originated from a rapid secretion from circulating leukocytes, a finding which also previously has been discussed [14]. The absence of a similar correlation for MPO makes it tempting to speculate that circulating MPO levels originated from other cellular sources than circulating leukocytes, for instance from stationary leukocytes embedded in the myocardium [34] or within the culprit lesion [35]. The significant correlation between both plasma PTX3 and MPO and neutrophil cell count, however, indicates that some release from neutrophil storage pools might be present, in accordance with existing literature [14, 33].

In line with some other reports [13, 15], though not all [36], we could not observe any correlation between PTX3 levels and LVEF or indices of myocardial injury. Interestingly, PTX3 levels have been reported elevated in heart failure with normal ejection fraction and diastolic dysfunction [37]. Baseline MPO levels, however, correlated inversely to baseline LVEF and positively to peak CKMB. In line with our observations, inverse correlation between peak MPO and functional recovery of infarcted myocardial regions [38] and preservation of left ventricular function after AMI in MPO-/- mice [11] has been reported. The pathophysiological mechanism behind the possible role of MPO as an actor in adverse ventricular remodeling and heart failure development after AMI has been suggested to be decreased inactivation of plasminogen activator inhibitor-1 (PAI-1) by MPO-generated oxidants with resultant decreased plasmin activity [11].

### Limitations

Assessment of PTX3 and MPO in relation to LVEF was not predefined endpoints in the ASTAMI trial, hence power calculation as to significant effect of mBMC on these spesific markers has not been performed. The median number of bone marrow cells injected (68 × 10^6^) could be discussed, as could also the timing of the intervention procedure [28].

### Conclusions

Administration of mBMCs after STEMI had limited effect on plasma levels of PTX3 and MPO. The initially high PTX3 and MPO levels, the genetic regulation of PTX3 in circulating leukocytes and the correlation between MPO and myocardial injury support the importance of neutrophil cell activation in STEMI.
